# Supplementary material for: Whole Genome Resequencing Identifies Single-Nucleotide Polymorphism Markers of Growth and Reproduction Traits in Zhedong and Zi Crossbred Geese
Source: Genes (Basel). 2023 Feb 14;14(2):487. doi: 10.3390/genes14020487 (PMC9956059; doi:10.3390/genes14020487)
Supplement: Supplementary file 1 [file genes-14-00487-s001.zip › genes-2140918-supplementary.pdf]

Certificate of English Language Editing

Manuscript Title:

Genome-wide association study identifies single-nucleotide polymorphism markers of growth and reproduction traits in Zhedong and Zi crossbred geese

Date of Revision:

February 3, 2023

Abstract:

The broodiness traits of domestic geese are a bottleneck that prevents the rapid development of the goose industry. To reduce the broodiness of the Zhedong goose and thus improve it, this study hybridized it with the Zi goose, which has almost no broody behavior. Genome resequencing was performed for the purebred Zhedong goose, as well as the F2 and F3 hybrids. The results showed that the F1 hybrids displayed significant heterosis in growth traits, and their body weight was significantly greater than those of the other groups. The F2 hybrids showed significant heterosis in egg-laying traits, and the number of eggs laid was significantly greater than those of the other groups. A total of 7,979,421 single-nucleotide polymorphisms (SNPs) were obtained, and three SNPs were screened. Molecular docking results showed that SNP11 located in the gene NUDT9 altered the structure and affinity of the binding socket. The results suggested that SNP11 is an SNP related to goose broodiness. In the future, we will use the cage breeding method to sample the same half-sib families to accurately identify SNP markers of growth and ...

This document certifies that the manuscript listed above was copy edited for English language by LetPub, with regard to grammar, punctuation, spelling, and clarity. All of our language editors are native English speakers with long-term experience in editing scientific and technical manuscripts. We are committed to leveling the playing field for researchers whose native language is not English.

- Documents receiving this certification should be regarded as having undergone professional editorial revision for English language before submission. However, the authors may accept or reject LetPub's suggestions and changes at their own discretion and LetPub does not have editorial control over the submitted documents.
- The language quality of the submitted document is the sole responsibility of the submitting authors subject to those authors' adherence to LetPub's revisions and instruction. LetPub's provision of service does not constitute a guarantee or endorsement of the authors' work herein.
- Neither the research content nor the authors' intended meaning were altered in any way during the editing process.
- If you have any questions or concerns about this edited document, please contact us at support@letpub.com

LetPub is an author service brand owned and operated by Accdon LLC. Headquartered in the Boston area, we are a full-spectrum author services company with a large team of US-based certified language and scientific editors, ISO 17001 accredited translators, and professional scientific illustrators and animators. We advocate ethical publication practices and are an official member of the Committee on Publication Ethics (COPE).

For more information about our company, services, and partnership programs, please visit www.letpub.com.

© 2023 Accdon, LLC. All Rights Reserved. Tel: 1-781-202-9968 Email: info@accdon.com Address: 400 Fifth Ave, Suite 530, Waltham, MA 02451, United States
